# Spontaneous closure of stoma

**DOI:** 10.1093/gastro/gov014

**Published:** 2015-05-05

**Authors:** Narendra Pandit, Harjeet Singh, Hemanth Kumar, Rajesh Gupta, G. R. Verma

**Affiliations:** Division of Surgical Gastroenterology Department of General Surgery, Post Graduate Institute of Medical Education and Research (PGIMER), Chandigarh, India

**Keywords:** ileostomy, colostomy, spontaneous closure of stoma

## Abstract

Intestinal loop stoma is a common surgical procedure performed for various benign and malignant abdominal problems, but it rarely undergoes spontaneous closure, without surgical intervention. Two male patients presented to our emergency surgical department with acute abdominal pain. One of them was diagnosed as having rectosigmoid perforation and underwent diversion sigmoid loop colostomy after primary closure of the perforation. The other was a known case of carcinoma of the rectum who had already undergone low anterior resection with covering loop ileostomy; the patient underwent second loop ileostomy, this time for complicated intestinal obstruction. To our surprise, both the loop colostomy and ileostomy closed spontaneously at 8 weeks and 6 weeks, respectively, without any consequences. Spontaneous stoma closure is a rare and interesting event. The exact etiology for spontaneous closure remains unknown, but it may be hypothesized to result from slow retraction of the stoma, added to the concept of a tendency towards spontaneous closure of enterocutaneous fistula.

## Introduction

An intestinal stoma is an artificial opening made in the small bowel or colon to divert flatus, faeces or urine outside the abdomen, where they are collected in external appliances [1]. Stomas can be temporary or permanent, depending on the purpose for which the diversion has been created. The most common indications are to protect a distal gastrointestinal anastomosis, to relieve a benign or malignant obstruction, or to control sepsis related to a perforation [2]. Once the indication is fulfilled, after the desired period of time, temporary stomas are closed surgically. Spontaneous closure of a stoma without surgical intervention is a rare entity. We present herein an interesting dual case of spontaneous closures of an ileostomy and a colostomy.

## Case presentation

### Case 1

A 64-year-old gentleman presented to surgical emergency with pain in the lower abdomen, constipation and fever of 2 days duration. Clinical examination showed tachycardia of 106 beats/min and blood pressure of 110/70 mmHg. Abdominal examination revealed mild abdominal distension with lower abdominal tenderness. Digital rectal examination was unremarkable.

**Figure 1.**
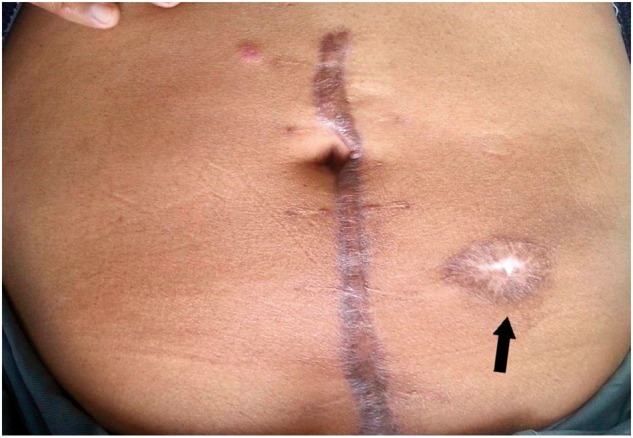
Site of loop colostomy (arrow), which underwent spontaneous closure. The skin is completely epithelialized.

Contrast-enhanced computed tomography (CT) of the abdomen with rectal contrast revealed mural thickening involving the rectosigmoid region with contrast extravasation, and pneumoperitoneum suggestive of rectosigmoid perforation. The patient underwent emergency exploratory laparotomy. Intraoperative findings revealed 500 mL of purulent fluid in the pelvis and a 1 × 1 cm perforation at the rectosigmoid junction on its anterior wall, with oedematous and thickened bowel wall. The perforation was closed with absorbable interrupted sutures. A proximal sigmoid loop colostomy was carried out. The perforation margin biopsy revealed non-specific inflammation. At a 2-month follow-up visit, the stoma was functioning, along with rectal passage of stool. The patient also had a retracted but well-functioning stoma, without any complaints. Three week later, the patient visited the outpatient department, exhibiting complete closure of the stoma, while passing stools rectally without any difficulty. Also the complete epithelialization of the stoma site was noted (Figure 1). Now, at 1-year follow-up, the patient is asymptomatic.

### Case 2

A 45-year-old gentleman presented with bleeding from the rectum and tenesmus that had lasted for 6 months. General and systemic examination was unremarkable. On digital rectal examination, an ulcerated mass was palpable, 6 cm from the anal verge. Biopsy showed moderately differentiated adenocarcinoma. Contrast-enhanced CT of the abdomen showed rectal malignancy with no distant metastases. The patient underwent laparoscopic low anterior resection with covering loop ileostomy. The post-operative period was uneventful and the patient was discharged on post-operative day 6.

Histopathological examination of a specimen showed adenocarcinoma of the rectum. The pathological stage of the tumour was T3N0M0 (Stage IIA). The patient received 5 Fluorouracil based adjuvant chemoradiotherpy with radiation dose of 45 Gy/25# over 4 week. One month after completion of chemoradiotherapy, the patient presented to the emergency surgical department with features of acute small bowel obstruction. He underwent exploratory laparotomy, which revealed an ileal loop, proximal to the covering loop ileostomy was densely adherent to pelvis which was the transition zone for the obstruction. The densely adherent bowel was difficult to release and, hence, another loop ileostomy was made on the left iliac fossa, 10 cm proximal to the site of obstruction.

**Figure 2.**
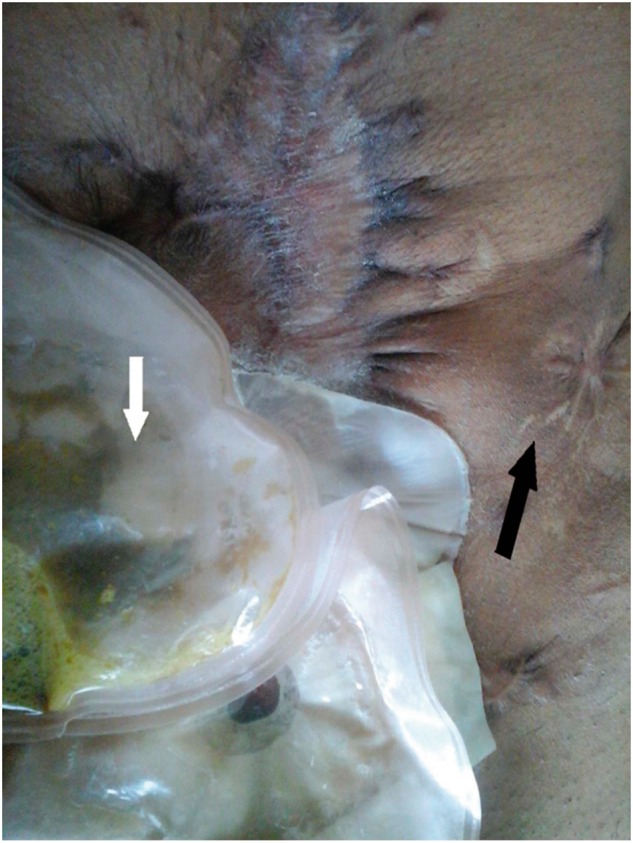
Site of loop ileostomy (black arrow), which closed spontaneously, with epithelialization of the skin, and the functioning covering loop ileostomy (white arrow).

Three weeks later, the right-sided covering loop ileostomy started functioning, with effluent also collecting partly in the left side stoma. The left stoma was gradually retracting and so was the effluent, with a corresponding increase of effluent in the right stoma. There was no intraperitoneal spillage of content. At 6 weeks following surgery there was complete retraction of the left stoma, with a completely functioning right-sided covering ileostomy. At 3-month follow-up, there was complete epithelialization of the left stoma site (Figure 2). Now, at 6-month follow-up, the patient is asymptomatic.

## Discussion

Intestinal stoma (ileostomy or colostomy) is a common surgical procedure performed for various benign and malignant problems. These stomas are generally created when the distal anastomosis or bowel closure is precarious or the patient is presenting with perforation peritonitis, where closure of perforation is risky. When the purpose of creating a stoma is achieved, it is closed but, in the interim period, some patients may develop stoma-related complications such as dehydration, necrosis and retraction [2].

Reports of spontaneous stoma closure are rarely found in the literature. We consider that these effects are probably due to the complications occurring with the stoma and we offer this insight into the mechanism of stoma closure by correlating the retraction of stoma and the process of healing of enterocutaneous fistula.

The overall incidence of retraction of a stoma—either an ileostomy or colostomy—ranges from 1.4–9.0%. It can develop either in the early post-operative period, or as a late complication [3]. These retracted loop stomas are problematical, because the stoma's ability to fully divert the faecal stream is compromised. Retraction in acute settings can result in dehiscence of the mucocutaneous junction and later subcutaneous and even intraperitoneal contamination. Retraction occurs because of excessive tension on the mesenteric side of the bowel, which usually occurs when there is inadequate mobilization of the bowel or oedema of the bowel and mesentery due to peritoneal inflammation, causing excessive tension on the stoma [4]. Moreover, retraction may develop due to mucocutaneous separation as a result of ischaemia, necrosis of the stoma, inadequate approximation of the mucosa to the dermal layer of skin, peristomal infection, or lack of maturation due to malnutrition. On the other hand, late retractions are known to occur through increased thickness of the abdominal wall with weight gain [5].

In both of our cases, spontaneous stoma closure can be explained by the mechanism of stoma retraction. In the spontaneous colostomy closure patient, it was the late retraction which added tension on the stoma, leading to gradual retraction with apposition and later fusion of the anterior stoma opening without any consequence. Similarly, in the second patient with spontaneous ileostomy closure, there was mucocutaneous separation—probably due to peristomal leakage and infection. This led to gradual retraction of the stoma and spontaneous complete closure of the ileostomy [6].

Another possible mechanism to explain this phenomenon may be seen as spontaneous closure of an enterocutaneous fistula; stomas are iatrogenic enterocutaneous fistulae. With conservative management, 19–92% of post-operative fistulae heal spontaneously, as long as there is no distal obstruction, the bowel is not diseased and the patient is in an anabolic state [7]. Even the more complex enteroatmospheric fistula, which occurs in the setting of open abdomen, has been reported to heal spontaneously with conservative management [8, 9]. So, in the present cases—considering the beneficial effects of retraction of stoma, with the concept of spontaneous healing of enterocutaneous fistula—the possible mechanism of spontaneous closure of stoma may be correlated and explained.

To summarize, spontaneous closure of a stoma is a rare event. The exact mechanism leading to closure is poorly understood; further studies, perhaps animal-based, may be required to obtain insight into the probable mechanism.

*Conflict of interest statement*: none declared.
